# In vitro and in vivo antiplasmodial activities of leaf extracts from *Sonchus arvensis* L.

**DOI:** 10.1186/s12906-023-03871-7

**Published:** 2023-02-14

**Authors:** Dwi Kusuma Wahyuni, Sumrit Wacharasindhu, Wichanee Bankeeree, Sri Puji Astuti Wahyuningsih, Wiwied Ekasari, Hery Purnobasuki, Hunsa Punnapayak, Sehanat Prasongsuk

**Affiliations:** 1 Plant Biomass Utilization Research Unit, Department of Botany, Faculty of Science, Chulalongkorn University, Bangkok, 10330 Thailand; 2 Department of Biology, Faculty of Science and Technology, Universitas Airlangga Surabaya, East Java, 60115 Indonesia; 3 Department of Chemistry, Faculty of Science, Chulalongkorn University, Bangkok, 10330 Thailand; 4 Department of Pharmaceutical Sciences, Faculty of Pharmacy, Universitas Airlangga, Surabaya, East Java 60115 Indonesia

**Keywords:** Antioxidant, Antiplasmodial, Hepatoprotective, Immunomodulator, Nephroprotective, Malaria, *Plasmodium berghei*, *Plasmodium falciparum*, *Sonchus arvensis* L.

## Abstract

**Background:**

Malaria continues to be a global problem due to the limited efficacy of current drugs and the natural products are a potential source for discovering new antimalarial agents. Therefore, the aims of this study were to investigate phytochemical properties, cytotoxic effect, antioxidant, and antiplasmodial activities of *Sonchus arvensis* L. leaf extracts both in vitro and in vivo.

**Methods:**

The extracts from *S. arvensis* L. leaf were prepared by successive maceration with *n*-hexane, ethyl acetate, and ethanol, and then subjected to quantitative phytochemical analysis using standard methods. The antimalarial activities of crude extracts were tested in vitro against *Plasmodium falciparum* 3D7 strain while the Peter's 4-day suppressive test model with *P. berghei*-infected mice was used to evaluate the in vivo antiplasmodial, hepatoprotective, nephroprotective, and immunomodulatory activities. The cytotoxic tests were also carried out using human hepatic cell lines in [3(4,5-dimethylthiazol-2-yl)-2,5-diphenyltetrazolium bromide] (MTT) assay.

**Result:**

The *n*-hexane**,** ethyl acetate, and ethanolic extracts of *S. arvensis* L. leaf exhibited good in vitro antiplasmodial activity with IC_50_ values 5.119 ± 3.27, 2.916 ± 2.34, and 8.026 ± 1.23 μg/mL, respectively. Each of the extracts also exhibited high antioxidant with low cytotoxic effects. Furthermore, the ethyl acetate extract showed in vivo antiplasmodial activity with ED_50_ = 46.31 ± 9.36 mg/kg body weight, as well as hepatoprotective, nephroprotective, and immunomodulatory activities in mice infected with *P. berghei*.

**Conclusion:**

This study highlights the antiplasmodial activities of *S. arvensis* L. leaf ethyl acetate extract against *P. falciparum* and *P. berghei* as well as the antioxidant, nephroprotective, hepatoprotective, and immunomodulatory activities with low toxicity. These results indicate the potential of *Sonchus arvensis* L. to be developed into a new antimalarial drug candidate. However, the compounds and transmission-blocking strategies for malaria control of *S. arvensis* L. extracts are essential for further study.

## Background

Malaria is an endemic disease in tropical areas of Asia, Africa, and Central and South America, and is known to spread even in vaccinated populations [1]. In 2020, malaria accounted for 241 million new infections and 627 thousand deaths worldwide in 87 endemic countries. There were 14 million more malaria cases and 47,000 more deaths compared to 2019 [2]. Therefore, one of the United Nations Millennium Development Goals (MDGs) is to reduce the incidence and subsequent morbidity and mortality associated with malaria [2]. The malaria elimination target in Indonesia is no high-endemic district by the end of 2024 [3]. However, the significant obstacles to the treatment and prognosis of malaria are its resistance to chloroquine [4, 5] and artemisinin-based combination therapy [2]. There is an urgent need to develop new antimalarial drugs and many researchers are currently exploring the efficacy of synthetic and natural products [6].

An estimated 80% of the global population uses natural products to treat various illnesses and diseases [7, 8]. In the case of malaria, 75% of patients have been reported to treat themselves with traditional medicines derived from various plant sources, including *Cinchona succirubra* L., as well as relatively newer medicines, such as artemisinin, which is produced from *Artemisia annua* L. [9]. In Indonesia, *Sonchus arvensis* L., a highly invasive species of the family Asteraceae, is used as a traditional medicinal plant for malaria treatment [10]. This plant contains various active compounds including flavonoids, saponins, and polyphenols [11], which have been reported for moderate to high antioxidant [12], hepatoprotective [13], nephroprotective [14], anti-inflammatory [15], and antibacterial activities [16–18]. Although *S. arvensis* L. has pharmaceutical benefits, it has never been evaluated for in vivo treatment of malaria.

The aim of the present study was to determine the in vitro and in vivo antiplasmodial activities of crude extracts from *S. arvensis* L. leaf, as well as the in vitro toxicity, in vitro antioxidant activities, and whole blood analysis of mice infected with *Plasmodium berghei*. The study results provide useful information regarding the antiplasmodial activity of a *S. arvensis* L. crude extract.

## Materials and Methods

### Plant collection and identification

*S. arvensis* L. was from Taman Husada Graha Famili (Medicinal Plant Graden of Graha Famili) Surabaya, East Java, Indonesia. The plant was cultivated in private field and harvested at 2–3 months before the generative stage. The leaves were green and healthy, with no indications of damage due to insects or microbes. The plant material used was confirmed by Mr. Dwi Narko, a botanist researcher at Purwodadi Botanical Garden, Indonesian Institute of Sciences, Purwodadi, East Java, Indonesia (number of determination 1020/IPH.3.04/HM/X/2019). The voucher specimen was deposited in the Plant Systematics Laboratory, Department of Biology, Faculty of Science and Technology, Universitas Airlangga (No. SA.0110292021).

### Plant extraction

The leaves of *S. arvensis* L. were air-dried and then ground into a powder (60-mesh size sieves). Each 1 kg of powder was separately macerated with different solvents including *n*-hexane, ethyl acetate, and ethanol for 24 h at room temperature (28 ± 2 °C) three times, filtered with filter paper (pore diameter 110 mm; Merck KGaA, Darmstadt, Germany), and then evaporated in a rotary evaporator at 60 °C to acquire crude extracts. The yields of the extracts (w/w) were measured prior to storage at 4 °C.

### Phytochemical screening

The crude extracts of *S. arvensis* L. leaf was screened for phytochemical content by standard methods including the Wilstatter "cyanidin" test for flavonoids, Mayer's test for alkaloids, the ferric chloride test for tannins, the Liebermann–Burchard test for terpenoids, and the foam test for saponins [19].

### Thin Layer Chromatography (TLC) Analysis

Five mg of each crude extract of *S. arvensis *L. leaf were dissolved in 100 µl of *n*-hexane, ethyl acetate, and ethanol, respectively. Aliquots of samples (5 µL; 250 µg) were spotted and allowed to dry on a TLC plate (Silica gel GF254). The plate was developed with *n*-hexane: ethyl acetate (4:1v/v) as the mobile phase. Detection of compounds was achieved by spraying with *ρ*-anisaldehyde sulfuric acid reagent [18], then heating at 105 °C for 10 min or until the colored bands appeared.

### Antioxidant assay

Antioxidant activity was evaluated by a method of 2,2-diphenyl-1-picryl-hydrazyl-hydrate (DPPH) assay based on a stable and synthetic radical [20]. When DPPH reacts with an antioxidant compound, its free radical property is lost and its color changes from violet to yellow. In brief, 100 µL of methanolic DPPH reagent (0.2 mM) was mixed with 100 µL of each sample in methanol at different concentrations (1.75, 3.15, 6.25, 10, 12.5, 15, 25, 35, 50, 75, 100, 150, and 200 μg/mL) and methanol as the control. The mixtures were incubated for 30 min in the dark at room temperature and the absorbance was measured at 517 nm. The assay was conducted in two independent wells for each sample and control for calculating the IC_50_ value and replicated three times_._ The DPPH radical scavenging capacity was calculated using the following equation:

1 $$\mathrm{DPPH}\;\mathrm{radical}\;\mathrm{scavenging}\;\mathrm{capacity}\;(\%)\hspace{0.17em}=\hspace{0.17em}[(\mathrm{A_{control}}-\mathrm{A_{sample}})/\mathrm{A}_{\text{control}}]\hspace{0.17em}\times\hspace{0.17em}100\%$$

where A_sample_ is the absorbance of the sample and A_control_ is the absorbance of the DPPH reagent at the wavelength of 517 nm. The results of DPPH radical scavenging capacity at different concentrations were then plotted and regressed linearly to obtain the IC_50_ values of samples. The IC_50_ value was calculated as the mean and standard deviation from triplicate samples.

### In vitro antiplasmodial activity assay

In this study, the antiplasmodial activity of leaf extracts was investigated against the chloroquine-sensitive strain of *P. falciparum* (3D7). The parasite was cultured in human O^Rh+^ red blood cells according to the method of Trager and Jensen [21] using Roswell Park Memorial Institute 1640 (RPMI-1640) medium supplemented with 50 µg/mL hypoxanthine, 2 mg/mL sodium bicarbonate (NaHCO_3_), 5.94 g/L of N-2-hydroxyethyl piperazine-N-2-ethane sulfonic acid (HEPES) and 10% serum blood group O^Rh+^. The parasitized culture suspension (1% parasitemia) was prepared in complete RPMI-1640 and 150-μL volume of this was dispensed in a 24-well microplate (5% hematocrit). The parasites were synchronized using 5% sorbitol to ring stage. The extracts and chloroquine diphosphate served as positive controls were dissolved in dimethyl sulfoxide (DMSO), diluted with medium to obtain the required concentrations (0.01, 0.1, 1, 10, and 100 μg/mL), and aliquoted [50 μL with final concentration at 0.5% (v/v) of DMSO] into each well of parasitized culture suspension. The parasitized cultures with 0.5% (v/v) of DMSO served as negative controls. The plates containing parasite cultures were incubated in an incubator (at 37 °C, 5% CO_2_, 95% humidity) for 48 h. The antiplasmodial assay of each extract were carried out in three replicates (n = 3). Afterward, the suspensions were collected, thinly smeared on glass slides, fixed with methanol, and stained with 10% Giemsa. The number of parasites was counted under a microscope and compared with the negative control to determine the extent of parasite growth inhibition in 5000 of total erythrocytes. The equation for calculating parasitemia, inhibition, and growth percentage used the equation method as described in a previous study [18].

The percentage of parasitemia was calculated using the formula:

2 $${\%\text{ Parasitemia}}=\frac{\sum \text{infected erythrocytes}}{\text{5000 of total erythrocytes}}\times \text{100}\% $$

The percentage of inhibition was counted using the equation:

3 $$\%\;\mathrm{Inhibition}=100\%-\left[\frac{\mathrm{Xp}}{\mathrm{Xk}}\times100\%\right]$$

The percentage of growth was calculated using the formula:

4 $$\%\;\mathrm{Growth}\;=\;\%\;\mathrm{parasitemia}\;\mathrm{Un}-\%\;\mathrm{parasitemia}\;\mathrm{Do}$$

Where:

Xp = Treatment parasitemia

Xk = Negative control parasitemia

Un = % parasitemia in each concentration

Do = % parasitemia at the start

∑ infected erythrocytes = the number of infected erythrocytes in 5000 of total erythrocytes

The probit analysis was conducted to calculate the IC_50_ values.

### Cytotoxicity test

Cytotoxicity of extracts was assessed by the method of 3- [4, 5-dimethylthiazol-2-yl] 2, 5-diphenyl tetrazolium bromide (MTT) assay as described by Fosenca et al. [22]. MTT assay detect the mammalian cells survival based on the tetrazolium salt signal when the tetrazolium ring is cleaved in active mitochondria [23]. Dimethyl sulfoxide (DMSO) was used to dissolve the extracts, then they were diluted with medium to obtain the required concentrations (6.25, 12.5, 25, 50, 100, 200, 400, 600, 800, and 1000 μg/mL). Human hepatic cell lines (Huh7it-1 cells) from Institute Tropical Diesases, Universitas Airlangga, Surabaya, Indonesia, were cultured in complete Dulbecco’s modified Eagle’s medium (DMEM) supplemented with 1% (v/v) glutamine (200 mM) at 37 °C under an atmosphere of 5% of carbon dioxide atmosphere and 95% humidity. The culture was conducted three times. The cell numbers were determined by measuring the absorbance at 560 nm and 750 nm using multiplate reader and the viability were assessed. The viability of cells was calculated using the equation:

5 $${\%\text{ of viability}}=(\mathrm{A_{sample}}/\mathrm{A}_{\text{control}})\times \text{100}\% $$

where A_sample_ was the absorbance sample at 560 nm-Absorbance sample at 750 nm and A_control_ was the absorbance DMEM medium. The percentage of cell viability was then plotted and regressed linearly to obtain the CC_50_ values. The selectivity index (SI) values were calculated based on the ratio between the CC_50_ value of cytotoxicity and antiplasmodial activity *P. falciparum* 3D7 from each extract (IC_50_).

### In vivo antiplasmodial activity assay

The extract with the highest antiplasmodial activity against *P. falciparum* 3D7 was selected to subsequently analyze the antiplasmodial activity against *P. berghei* (the mice-infected Plasmodium) with Peter’s method [24]. The strain of *P. berghei* ANKA was obtained from the Eijkman Institute of Molecular Biology (Jakarta, Indonesia). Blood infected with *P. berghei* ANKA was taken from mice with 20% parasitemia and diluted with phosphate-buffered saline. Swiss mice *Mus musculus* of BALB/c strain (male; body weight 25 ± 3 g; 6–8 weeks old) were intraperitoneally injected with 0.2 mL blood (1 × 10^6^ ANKA parasitized erythrocytes) and randomly divided (n = 7 per group) into four experimental groups and three control groups (normal, negative, and positive control). The experimental groups were orally treated with 0.25 mL single dose of 1, 10, 100, or 200 mg/kg BW of leaf extract (in 0.5% sodium carboxymethyl cellulose (Na-CMC)) two times per day for four days for antiplasmodial assay and continued seven days for biochemical analysis. The negative and positive control groups were treated with 0.5% Na-CMC suspension and 10 mg/kg BW of chloroquine diphosphate, respectively. The normal control group was the uninfected and untreated mice group (Table 1). On each day, blood was collected from the tail vein of each mouse, thinly smeared on a glass slide, fixed with methanol, and then stained with Giemsa. The slides were then observed under a microscope to calculate the percentage of parasitemia, inhibition, and growth. The formula of them used as described in in vitro antiplasmodial activity against *P. falciparum* 3D7. The median effective dose or effective dose for 50% of the population (ED_50_) was calculated with Probit analysis. The ED_50_ was calculated from each replication, and then averaged getting the mean and deviation standard.

**Table 1:** Experimental design of in vivo study for antiplasmodial activity and biochemical analysis

Experiment Group	*Plasmodium berghei-* infected mice	Treatment	Days	Replication
Normal Control (healthy/untreated)	-	Na-CMC	7	7
Positive Control	+	Chloroquine-phosphate	7	7
Negative Control	+	Na-CMC	7	7
T1	+	1 mg/kg BW	7	7
T2	+	10 mg/kg BW	7	7
T3	+	100 mg/kg BW	7	7
T4	+	200 mg/kg BW	7	7

Mice blood sample was analyzed for the percentage of parasitemia for 4 days except for the normal control and biochemical analysis on the seventh day for all treatment and control

## Biochemical analysis

After seven-day treatments, blood samples (0.5–0.75 mL) were collected from the left ventricle of each mouse into 1.5-mL microtubes and left standing at room temperature for 2 h. Then, serum was isolated by centrifugation at 3000 rpm for 20 min. The levels of serum glutamic-oxaloacetic transaminase (SGOT) and serum glutamic-pyruvic transaminase (SGPT) in obtained serum were measured using commercial enzyme-linked immunosorbent assay (ELISA) kits (DiaSys Diagnotic System, Holzheim, Germany) to assess the hepatoprotective effects of the selected extract on infected mice. For analyzing nephroprotective effects, blood urea nitrogen (BUN) and creatinine levels were measured using commercial ELISA kits (DiaSys Diagnotic System, Holzheim, Germany). The level of tumor necrosis factor-alpha (TNF-α) and interleukin 10 (IL-10) in serum was also analyzed to investigate the immune response of treated/control mice using commercial ELISA kits (BioLegend, San Diego, CA, USA). The replication of samples was four to seven times (Table 1).

### Data analysis

Data are expressed as the mean ± standard deviation (SD). The IC_50_ of antioxidant and CC_50_ of cytotoxicity were counted using regression linearly (Microsoft Excel). The Probit analysis was conducted to calculate the IC_50_ and ED_50_ values. Statistical significance was determined with the one-way analysis of variance (ANOVA) continued with Duncan Multiple Range Test (DMRT) for IL10 and TNFα, with a nonparametric independent *t*-test for SGOT and SGPT, and Kruskal–Wallis continued with Mann Whitney test for BUN and creatinine data. The level of significance was set at 0.05. All statistical analyses were conducted using IBM SPSS Statistics for Windows, version 20.0. (IBM Corporation, Armonk, NY, USA).

## Results

### The extract yields and phytochemical screening

The dried leaf was successively macerated and each kilogram of dried leaf yielded 59.26 ± 2.04 g of ethanol extract, 10 ± 1.1 g of ethyl acetate extract, and 25.9 ± 5.5 g of *n*-hexane extract (Table 2).

**Table 2:** Extraction yield of *Sonchus arvensis* L. leaf

**No**	**Extract**	**Yield (**g/kg)
1	*n*-Hexane	25.90 ± 5.50
2	Ethyl acetate	10.00 ± 1.10
3	Ethanol	59.26 ± 2.04

The data were represented as mean ± standard deviation (SD), *n* = 3

Secondary metabolites including terpenoids, flavonoids, and alkaloids were present in all extracts. However, saponins and polyphenols were present only in the ethanol and ethyl acetate extracts respectively (Table 3).

**Table 3:** Phytochemical screening of *Sonchus arvensis* L. leaf extract

No	Phytochemical agent	*n*-Hexane	Ethyl acetate	Ethanol
1	Terpenoids	+ +	+ + +	+
2	Flavonoids	+	+ +	+ +
3	Alkaloids	+	+	+
4	Saponin	-	-	+ +
5	Polyphenol	-	+	+ +

+  + , Strongly positive; + , Weakly positive; − , Not detected

### Terpenoid screening of the extracts of *Sonchus arvensis* L. by thin-layer chromatography (TLC)

**Fig. 1:**
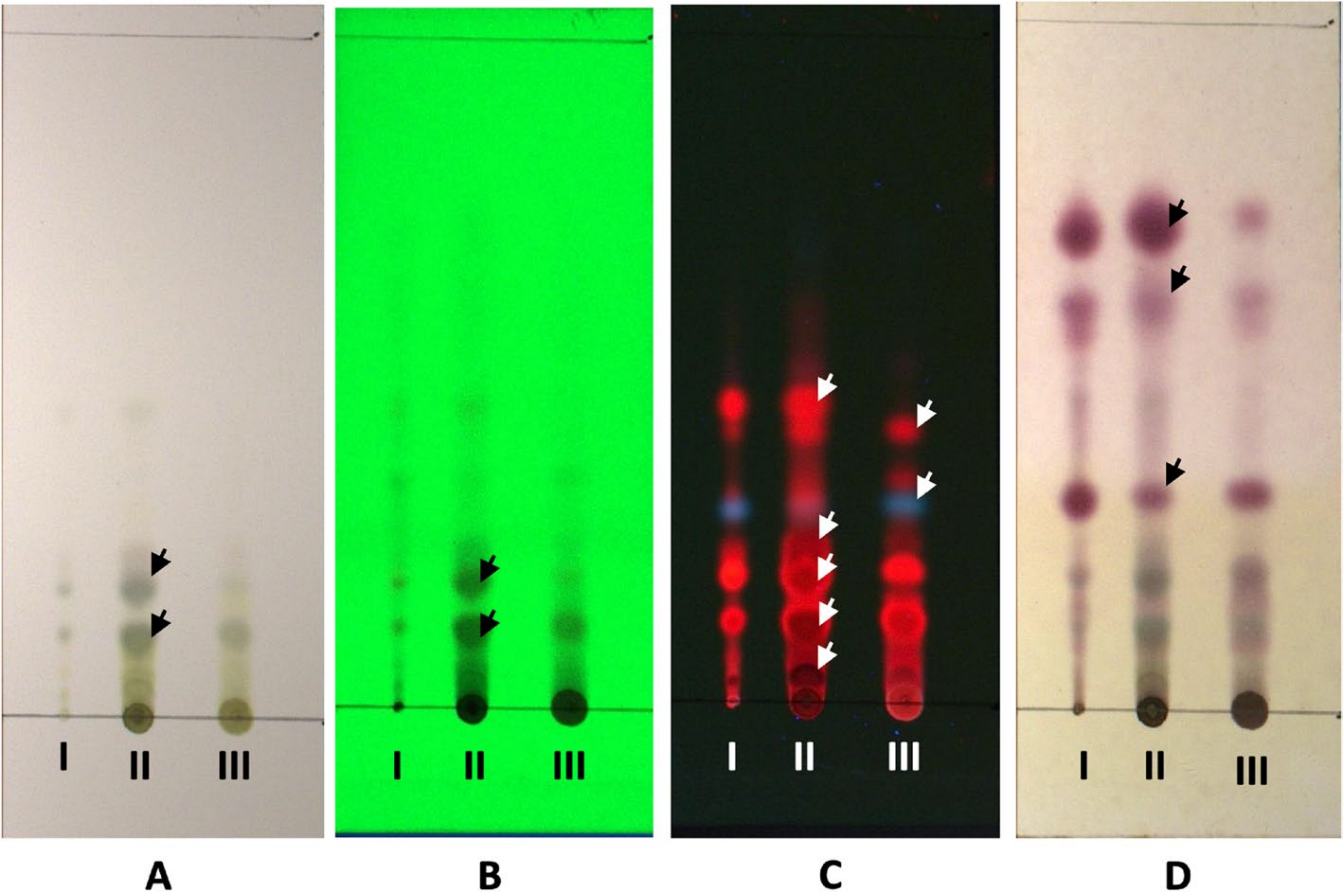
Chromatogram of *Sonchus arvensis* L. extracts TLC. **A**. Day light, **B**. UV 254 nm, **C**. UV 366 nm, **D**. After spray with *p*-anisaldehyde sulfuric acid (the purple spot is terpenoid), I. *n*-Hexane extract, II. Ethyl acetate extract, III. Ethanol extract

The *n*-hexane, ethyl acetate, and ethanol extracts of *Sonchus arvensis* L. were observed by TLC and there were two visible spots in daylight and under 254-nm UV light (R_f_ value = 0.12 and 0.18). Under 366-nm UV light, there were five separate spots with R_f_ values of 0.14, 0.19, 0.24, 0.35, and 0.53. After staining using *p*-anisaldehyde sulfuric acid, three separate purple stains were seen, with R_f_ values of 0.31, 0.59, and 0.71 (Fig. 1).

**Fig. 2:**
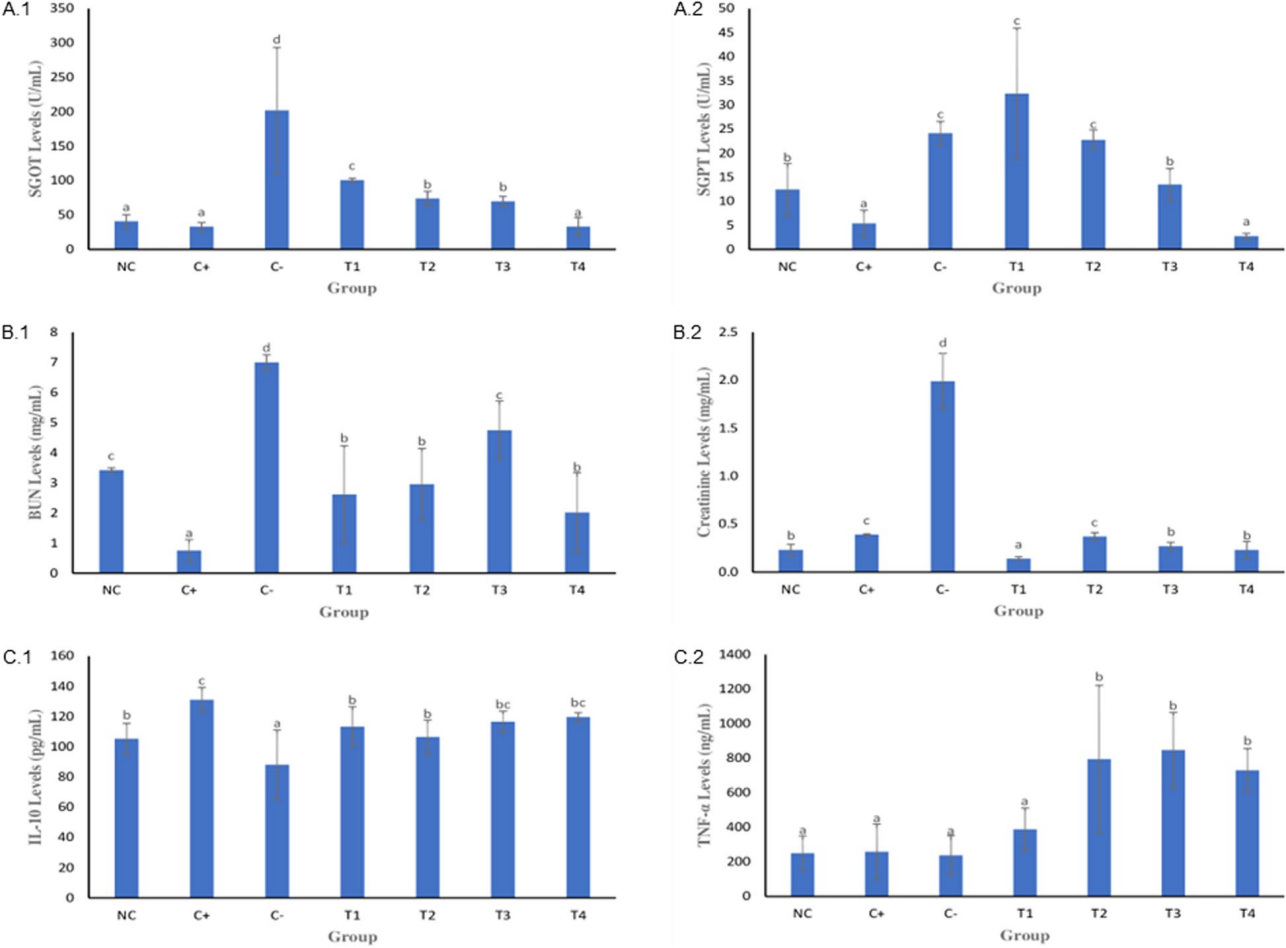
Serum SGOT, SGPT, BUN, creatinine, IL-10, and TNFα levels of mice infected with *P. berghei* strain ANKA after treatment with *S. arvesis* L. leaf ethyl acetate extract. SGOT (A.1); SGPT (A.2); BUN (B.1); creatinine (B.2); TNFα (C.1); TNFα IL-10 (C.2); CN: normal control (healthy/untreated) goup; C + : positive control; C-: negative control; T1: 1 mg/kg; T2: 10 mg/kg; T3: 100 mg/kg; T4: 200 mg/kg. The values followed by the same letter (superscripts) show no significant difference in the one-way analysis of variance (ANOVA) continued with Duncan Multiple Range Test (DMRT) for IL10 and TNFα, with a nonparametric independent *t*-test for SGOT and SGPT, and Kruskal–Wallis continued with Mann Whitney test for BUN and creatinine data. The significance level was set at 0.05

### Antioxidant activities

The DPPH assay was conducted to assess antioxidant activities. The IC_50_ values of all extracts are shown in Table 4. All extracts possessed antioxidant activities. From lowest to highest, the IC_50_ values were 8.27 ± 4.93, 12.36 ± 10.40, 31.35 ± 3.27, and 108.59 ± 11.24 µg/mL for the ethyl acetate, ethanol, methanol, and *n*-hexane extracts, respectively. Furthermore, the IC_50_ of ascorbic acid as standard was 22.63 ± 1.40 µg/mL.

**Table 4:** In vitro antioxidant activity of *Sonchus arvensis* L. leaf extract

No	Extract	Antioxidant activity (IC_50_, µg/mL)
1	*n*-Hexane	108.59 ± 11.24
2	Ethyl acetate	8.27 ± 4.93
3	Ethanol	12.36 ± 10.40
4	Methanol	31.35 ± 3.27
5	Ascorbic acid	22.63 ± 1.40

The data were represented as mean ± standard deviation (SD), *n* = 3

### In vitro antiplasmodial activity

The IC_50_ values of all extracts (Table 5) of *Sonchus arvensis* L. leaf at various doses indicated that the ethyl acetate extract (IC_50_ 2.92 ± 3.27 μg/mL) had the highest in vitro antimalarial activity, followed by the *n*-hexane and ethanol extracts.

**Table 5:** In vitro antiplasmodial activity of *Sonchus arvensis* L. leaf extracts against *P. falciparum* strain 3D7

No	Extract	% Inhibition at each concentration (μg/mL)						IC_50_ (µg/mL)
		**100**	**10**	**1**	**0.1**	**0.01**	**0.001**	
1	*n*-Hexane	100.00 ± 0.00	53.81 ± 0.65	45.21 ± 1.94	35.79 ± 3.90	31.73 ± 3.73	-	5.12 ± 2.34
2	Ethyl acetate	100.00 ± 0.00	75.82 ± 1.93	65.68 ± 3.09	44.89 ± 9.73	34.84 ± 1.49	-	2.92 ± 3.27
3	Ethanol	93.60 ± 0.53	27.54 ± 2.51	23.50 ± 1.11	13.09 ± 1.49	6.94 ± 0.36	-	8.03 ± 1.23
4	Chloroquine diphosphate	100.00 ± 0.00	100.00 ± 0.00	100.00 ± 0.00	79.76 ± 4.51	40.49 ± 5.29	17.17 ± 2.31	0.01 ± 0.00

Note: The data were represented as mean ± standard deviation (SD), *n* = 3

### Toxicity and selectivity index (SI)

The toxicity of all *Sonchus arvensis* L. extracts to hepatocytes was determined. From highest to lowest, the CC_50_ values of the extracts were 1420.88 ± 20.88, 437.39 ± 7.46, and 778.77 ± 10.53 µg/mL for *n*-hexane, ethanol, and ethyl acetate extract, respectively (Table 6). Then, the SI was calculated by comparing the toxicity and in vitro antimalarial activity. From highest to lowest, the SI values were 277.57 ± 5.77, 150.00 ± 3.62, and 97.03 ± 13.13 for *n*-hexane, ethanol, and ethyl acetate extract, respectively (Table 6).

**Table 6:** In vitro toxicity and selectivity index (SI) of *Sonchus arvensis* L. leaf extract

No	Extract	In Vitro Toxicity, CC_50_ (µg/mL)	Selectivity Index (SI)
1	*n*-Hexane	1420.88 ± 20.88	277.57 ± 5.77
2	Ethyl Acetate	437.39 ± 7.46	150.00 ± 3.62
3	Ethanol	778.77 ± 10.53	97.03 ± 13.13

Note: The data were represented as mean ± standard deviation (SD), *n* = 3

### In vivo antiplasmodial activity

The in vivo antimalarial activity of the ethyl acetate extract was also determined. As shown in Table 7, the parasitemia rates decreased with increasing doses. The inhibition of % parasitemia at doses at 1, 10, 100, and 200 mg/kg BW were 0%, 32.86 ± 7.50%, 56.32 ± 2.64% and 77.48 ± 2.93%, respectively. Probit analysis determined that the ED_50_ value of the ethyl acetate extract was 46.31 ± 9.36 mg/kg BW. This result was significant as compared to the negative control.

**Table 7:** Parasitemia, growth, and inhibition percentage of *Sonchus arvensis* L. leaves ethyl acetate extract against *Plasmodium berghei*

Sample	Dose (mg/kg)	Mean % Parasitemia		Mean % growth	Mean % inhibition	ED_50_ (mg/kg)
		Day 0	Day 4			
Ethyl acetate extract of *S. arvensis* L. leaves	1	1.51 ± 0.07	7.44 ± 0.21	5.93 ± 0.17	ND	46.31 ± 9.36
	10	1.42 ± 0.07	5.39 ± 0.43	3.97 ± 0.44	32.86 ± 7.50	
	100	1.51 ± 0.07	5.09 ± 0.56	2.57 ± 0.12	56.32 ± 2.64	
	200	1.46 ± 0.09	3.96 ± 0.68	1.36 ± 0.12	77.48 ± 2.93	
Na-CMC	-	1.44 ± 0.12	7.36 ± 0.24	5.91 ± 0.31	ND	
Chloroquine diphosphate	10	1.48 ± 0.09	0.02 ± 0.01	ND	ND	

Noted: The data have been represented as mean ± standard deviation (SD), n = 7. The Na-CMC: sodium carboxymethyl cellulose. *ND* = not detected

### Whole blood analysis of *P. berghei* infected mice

Mice infected with *P. berghei* were orally administered ethyl acetate extract of *S. arvensis* L. leaves two times per day for 7 days. Afterward, blood samples were collected to determine the hepatoprotective (SGOT and SGPT), nephroprotective (BUN and creatinine), and immunomodulatory (IL-10 and TNFα) effects (Fig. 2).

### SGOT and SGPT levels

The liver cells will spill the enzymes including aspartate aminotransferase (SGOT) and alanine aminotransferase (SGPT) into the blood to respond with liver injury. Therefore, raising the enzyme levels in the blood is the signaling to indicate the damage of liver. As compared to the negative control (201.87 ± 91.73 Ug/mL), serum SGOT levels of mice infected with *P. berghei* were significantly decreased by treatment with ethyl acetate extract at 1, 10, 100, and 200 mg/kg BW (100.68 ± 2.98, 73.85 ± 10.41, 69.82 ± 7.10, and 33.11 ± 13.16 Ug/mL, respectively, *p* < 0.05). However, there was no different significant between normal control (40.76 ± 9.59 U/mL), positive control (32.92 ± 6.07 U/mL), and treatment groups (31.11 ± 13.16 U/mL at a concentration of 200 mg/kg BW) (Fig. 2.A.1).

Furthermore, as compared to negative control (24.13 ± 2.45 U/mL) and treatment with ethyl acetate extract at 1 mg/kg BW (32.37 ± 13.6 U/mL) and 10 mg/kg BW (22.74 ± 2.08 U/mL), serum SGPT levels were significantly decreased in the groups treated with 100 and 200 mg/kg BW (13.45 ± 3.4 and 2.75 ± 0.59 U/mL, respectively) as well as the positive and normal control groups (5.4 ± 2.73 and 12.4 ± 0.84 U/mL, respectively). Overall, the SGOT and SGPT levels significantly decreased in the treatment groups compared with the negative control group (*p* < 0.05) (Fig. 2.A.2).

### BUN and creatinine levels

Relatively higher serum levels of BUN and creatinine are indicative of severe renal injury. As compared to the negative control (*Plasmodium berghei* infected mice that was given Na-CMC) group (7.00 ± 0.26 mg/mL), the serum BUN levels of the infected mice were significantly decreased by treatment with the ethyl acetate extract at 1, 10 100, and 200 mg/kg BW (2.61 ± 1.62, 2.95 ± 1.19, 4.75 ± 0.97, and 2.01 ± 1.33 mg/mL, respectively), *p* < 0.05 (Fig. 2.B.1). In addition, as compared to the negative control group (1.99 ± 0.29 mg/mL), serum creatinine levels were significantly decreased by treatment with the ethyl acetate extract at 1, 10, 100, and 200 mg/kg BW (0.14 ± 0.02, 0.37 ± 0.04, 0.27 ± 0.04, and 0.23 ± 0.06 mg/mL, respectively), *p* < 0.05 (Fig. 2 .B.2).

### Cytokine production

Serum IL-10 and TNFα levels were significantly increased in the treatment groups as compared to the normal and negative control groups. As compared to the negative control group (88.00 ± 22.96 pg/mL), serum IL-10 levels were significantly increased in the positive control group (131.09 ± 8.13 pg/mL) and slightly increased in the groups treated with the ethyl acetate extract at 1, 10, 100, and 200 mg/kg BW (113.27 ± 13.21, 106.36 ± 11.32, 116.55 ± 6.95, and 119.64 ± 2.84 pg/mL, respectively, *p* < 0.05), while there was no significant difference as compared to the normal control group (Fig. 2 .C.1).

Moreover, as compared to treatment with the ethyl acetate extract at 1 mg/kg BW (387.93 ± 123.59 ng/mL), serum levels of TNFα were significantly increased by treatment with 10, 100, and 200 mg/kg BW (794.93 ± 427.89, 848.07 ± 216.86, and 729.64 ± 126.89 ng/mL, respectively, *p* < 0.05). There were also significant differences among the negative, normal control, and positive control groups (237.64 ± 113.123, 249.64 ± 99.97, and 257.93 ± 160.33 ng/mL, respectively, *p* < 0.05). Collectively, these results suggest that ethyl acetate extract of *S. arvensis* L. enhances the immune response of mice against *P. berghei* infection (Fig. 2 .C.2).

## Discussion

Management and accessibility of healthcare are important problems in Eastern Indonesia. Hence, home remedies with traditional medicines is the most common method for the treatment of malaria. The use of traditional medicine is safe, cost-effective, and efficient. According to the World Health Organization (WHO), the use of traditional medicines continues to increase worldwide. Traditional medicines are rooted in Indonesian culture and history, although many traditional treatments have not been scientifically validated. Among the strategic objectives proposed by the WHO, the safety and efficacy of traditional medicines are primary goals before integrating traditional drugs in modern healthcare [2].

*S. arvensis* L. is the seventh most popular medicinal plant for treating various diseases in Indonesia, especially in Java and Bali [12]. Although the extract of *S. arvensis* L. callus is reported to possess antiplasmodial activities [17, 18], the efficiency and safety for malaria treatment have not been registered. Hence, the aim of this study was to evaluate the antiplasmodial actvity, toxicity, and antioxidant activity of crude extracts of *S. arvensis* L. leaf.

One kilogram of dried *S. arvensis* L. leaf was extracted by successive maceration with *n*-hexane, ethyl acetate, and ethanol. From each solvent, different extract weights were obtained. Each of the *S. arvensis* L. extracts was screened for the presence of phytochemicals. The ethanol extract contained flavonoids, alkaloids, terpenoids, saponins, and polyphenols, while the ethyl acetate extract included flavonoids, alkaloids, terpenoids, and polyphenols, and the *n*-hexane extract contained flavonoids, alkaloids, and terpenoids.

The Wilstatter "cyanidin" test confirmed the presence of flavonoids, while testing of the extract showed the presence of alkaloids, as indicated by the formation of a white precipitate after the addition of Mayer reagent. The Liebermann–Burchard test results confirmed the presence of terpenoids, as indicated by the yellow color of the solution. After adding a few drops of 10% FeCl_3_, the color of the solution changed to dark green, indicating the presence of tannins. Meanwhile, the presence of saponins was confirmed if the foam extract did not disappear after the addition of distilled water and shaking [11].

Polyphenols were present in the ethanol extract, whereas relatively large amounts of terpenoids were confirmed in the ethyl acetate and *n*-hexane extracts. These findings are consistent with similar studies conducted by Khan [25] and Seal [26]. Many triterpenoids have been isolated from the *n*-hexane extract of *S. arvensis* L. [16]. Additionally, some phytochemicals may be responsible for the various activities of *S. arvensis* L. For example, flavonoid and phenolic compounds possess antioxidant activities [25], saponins have anti-inflammatory activities [27], and terpenoids exhibit antimicrobial activities [16].

Furthermore, *S. arvensis* L extract showed antioxidant activities. The ethyl acetate and ethanol extracts exhibited potent antioxidant activities (IC_50_ < 50 µg/mL) [28], with IC_50_ value 8.27 ± 4.93 µg/mL and 12.36 ± 10.40 µg/mL respectively. While *n*-hexane extract had moderate antioxidant activity (101 > IC_50_ < 250 µg/mL) [28]. The IC_50_ value (the antioxidant activity) of ethyl acetate extract was lower than ascorbic acid as standard (22.63 ± 1.40 µg/mL) (Table 3). Moreover, compared to the other studies, the leaf extract from *S. arvensis* L was lower than those of plants and callus *Trifolium pratense* L. [29], *Callisia fragrance* leaf juice [30], and *Centella asiatica* L. leaf [31] that reported has antioxidant activity. The potent antioxidant activity of the *S. arvensis* L. extract was probably due to the presence of active ingredients with antioxidant activities, such as polyphenols and flavonoids. These findings are similar to those of previous studies of different plant sources [15, 25, 26, 32].

The antioxidant activity of the ethyl acetate extract was as good as the in vitro antimalarial effect. In general, an IC_50_ value less than 10 μg/mL is considered to indicate the high activity, while 10 < IC_50_ ≤ 25 μg/mL can be regarded as moderately active and values > 25 μg/mL are deemed inactive [33, 34]. All plant extracts in this study presented the IC_50_ values < 10 μg/mL; therefore, they were primarily considered as the new candidates for antimalarial-drug development. The IC_50_ values of the ethyl acetate extract,* n*-hexane, and ethanol extracts were 2.916, 5.119, and 8.026 μg/mL, respectively. The industry standardizes the IC_50_ value of in vitro antiplasmodial activity, a pure compound is said to be active as antiplasmodial activity if the IC_50_ value is below 10 μg/mL [6].

The toxicity of *S. arvensis* L. extracts were evaluated by calculating the ratio of cytotoxicity with human hepatic cell lines (CC_50_) to in vitro antiplasmodial activity expressed as IC_50_ (selectivity index SI = CC_50_/IC_50_). A higher SI, theoretically, indicates greater drug effectiveness and safety for the treatment of plasmodial infections. An ideal drug would be cytotoxic only at very high concentrations and have antiplasmodial activities at low concentrations, thus yielding a high SI value and eliminating the plasmodial target at concentrations well below the cytotoxic concentration [35]. The IC_50_ values of extracts toxicity were 1420.88 ± 20.88, 778.77 ± 10.53, and 437.39 ± 7.46, μg/mL, and then the SI value were 277.57 ± 5.77, 97.03 ± 13.13, and 150 ± 3.62 for *n*-hexane, ethanol, and ethyl acetate extract respectively. de Souza et al. [36] mentioned that “the natural product has been suggested that the SI > 10 indicate a favorable safety window between the effective concentration against the parasite and the toxic concentration to human cell”. So, all *S. arvensis* L.leaf extracts exhibited low toxicity. Nurianti et al. [37] found that an ethyl acetate extract of *S. arvensis* L had no toxic effects, and Harun et al. [38] revealed that an ethanol extract of *S. arvensis* L. was not toxic to healthy male albino rats. The antioxidant activities suggest that these extracts are relatively non-toxic because oxidative stress represents an imbalance between the production of free radicals and the ability of a biological system to readily detoxify reactive intermediates or repair the resulting damage [39].

Moreover, the ethyl acetate extract of *S. arvensis* L. was chosen for the assessment of in vivo antiplasmodial activity because it exhibited the highest antioxidant and in vitro antiplasmodial activities, with IC_50_= 8.27 and 2.92 μg/mL respectively. In vivo antiplasmodial activity can normally be classified as moderate, good, and very good if an extract displayed percentage inhibition equal to or greater than 50% at a dose of 500, 250, and 100 mg/kg BW per day, respectively [40]. *P. berghei*-infected mice given orally 50–250 mg/kg/day of extract exhibiting inhibition percentage > 60% are considered to be active or very active, and those exhibiting inhibition percentage > 30% are considered to be moderately active [33, 34]. Based on this classification, the ethyl acetate extract of *S. arvensis* L. showed excellent in vivo antiplasmodial activity below 100 mg/kg/day with an ED_50_ of 46.31 ± 9.36 mg/kg. The ED_50_ of ethyl acetate extract of *S. arvensis* L. was higher than the ethanolic extract of *H. annuus* root has an ED_50_ value of 10.6 ± 0.2 mg/kg [41] but lower than the *Tagetes erecta* L. and *Synedrella nodiflora* (L.) Gaertn. extract can significantly suppress parasitemia in malaria-infected mice by 50.82% and 57.67% respectively at 400 mg/kg BW dose [40]. Compared to another Asteraceae member, the ethyl acetate extract of *S. arvensis* L. could be developed as an antiplasmodial agent.

Furthermore, blood was collected from the experimental mice to determine the nephroprotective, hepatoprotective, and immunomodulatory activities after 7 days of treatment with ethyl acetate extract of *S. arvensis* L. Many studies have reported that *S. arvensis* L. extracts exhibited antioxidant [25], hepatoprotective [13], nephroprotective [42], and immunomodulatory [43] activities. The present study was conducted to assess the effect of an ethyl acetate extract of *S. arvensis* L. against *P. berghei* infection in mice*.*

An increase in SGPT and SGOT serum levels indicates liver damage [15], and a rise in BUN and creatinine levels suggests a failure of the kidneys or their possible malfunction [39]. The results showed that the ethyl acetate extract protected the liver and kidneys by reducing SGOT, SGPT, creatinine, and BUN levels.

Overall, the serum levels suggested that the ethyl acetate extract of *S. arvensis* L showed nephroprotective, hepatoprotective, and immunomodulatory activities in mice infected with *P. berghei.* The result is very interesting because the pathogenesis caused by *P. berghei* is multifactorial and has not been well characterized. There were several hypotheses suggesting that erythrocyte cytoadherence, proinflammatory response, nephrotoxicity, and oxidative stress are involved in the pathogenesis of *P. berghei* [44, 45]. Free heme-mediated oxidative stress, in which free heme is produced by parasites that consume hemoglobin during the intra-erythrocytic phase, has been implicated in lipoprotein oxidation and serious kidney damage [46]. In addition, malaria infection is caused by parasites and host factors, where there will be microvascular disturbances in the host's body. *P. berghei* parasites will infect erythrocytes and activate cytokines of phagocytic cells and endothelial cells to produce TNF-α, IL-10, IFN-γ, and free radicals (ROI, ROS, and NO). Free radicals are molecules with one unpaired electron in their outer orbit which makes the molecule unstable [42]. Free radicals can cause oxidative stress. It has implications for various pathological conditions [48]. The involvement of oxidative stress can cause the amount of antioxidant status to decrease [42]. Oxidative stress condition is defined as imbalance condition between antioxidants and free radicals, where the state of free radicals is higher than antioxidants [14]. The number of antioxidants decreases because the body used to balance the high free radicals due to the presence of parasites. The more severe the infection from *P. berghei*, the use of antioxidants in the body will increase, causing the number of antioxidants in the body to decrease [42]. The biochemical data of serum of mice infected *P. berghei* supported that the *S. arvensis* L. ethyl acetate extract is active as antiplasmodial. Particularly, the *S. arvensis* L. leaf ethyl acetate extract increases the mice immune response to *P. berghei* infections. It is very valuable for the further investigation of the in vivo antioxidant activity and cytotoxicity of the *S. arvensis* L. leaf as antiplasmodial drug candidate.

The comprehensive tests and discussions in this study confirmed that ethyl acetate extract possessed antiplasmodial both in vitro and in vivo with nephroprotective, hepatoprotective, and immunomodulatory activities in mice infected with *P. berghei*. This study highlighted that *S. arvensis* L. crude extract had antimalarial activity. In summary, the results suggest that ethyl acetate extract of *S. arvensis* L. could be used to develop new antimalarial drugs in the future from a natural resource.

## Conclusion

The results of this study confirmed the antiplasmodial activity of ethyl acetate extract of *S. arvensis* L. both in vitro and in vivo as well as the antioxidant, nephroprotective, hepatoprotective, and immunomodulatory activities with low toxicity. It was strongly suggesting the potential as an antimalarial drug. These findings lay a foundation for further investigations of new antimalarial compounds for future pharmaceutical applications. Further research, including bioassay-guided fractionation, was also recommended to identify new antimalarial drug candidates.

## Data Availability

The datasets generated and analyzed during the current study are not available online due to funding policy however they are available from the corresponding authors or first author on reasonable request.

## Abbreviations

BUN: Blood urea nitrogen
SGOT: Serum glutamic oxaloacetic transaminase
SGPT: Serum glutamic pyruvic transaminase
TNFα: Tumor necrosis factor alpha
IL-10: Interleukin 10
IC_50_: Half maximal inhibitory concentration
ED_50_: Median effective dose
DPPH: 2,2-Diphenyl-1-picryl-hydrazyl-hydrate
MTT: 3-(4,5-Dimethylthiazol-2-Yl)-2,5-Diphenyltetrazolium Bromide
